# Practical Methods in Porcelain Fillings

**Published:** 1903-06-15

**Authors:** Henry C. Raymond


### PRACTICAL METHODS IN PORCELAIN FILLINGS.

BY HENRY C. RAYMOND, D.D.S.

Read before the Annual Meeting of the Central Michigan Dental Association, May 13, 1903.

It is not my intention to take up your time with incomplete descriptions of the several processes employed in porcelain filling, but rather to try and put before you, in as plain a manner as possible, the method employed by those who have achieved the greatest success in this beautiful art, a method that seems to me the simplest and most practical, and from which I have obtained most satisfactory results.

Probably there are some here who are familiar with the work, and for them my paper may not contain much that is new. They will, however, be the better qualified to discuss its contents, and so bring out points that I may miss. To those of you who know little or nothing of the work, but who are interested in it, as certainly all should be, is my paper principally addressed, and if I can succeed in making myself understood in explaining the technic of porce'ain fill ngs, I will feel that I have served a good purpose.

In bringing up or advocating any new process or material for our consideration, the operator, as much as the patient, wishes to know how practical and permanent it is, and to what extent it may be employed. Although porcelain filling as a process is not new, in its present stage of development, with the materials and appliances now at hand, it is certainly not old. In fact, I think I may say that to the majority of practitioners it is almost a closed book.

It will be proper, therefore, for me first to state the cases in which it may be successfully used, and in this connection I might say that if the operator has the ability, there are few teeth that cannot be successfully filled with porcelain. You will note that I say *successfully* filled; by that I mean filled so as to preserve the teeth, and that, too, as well or better than such teeth can be preserved with any other material. This, I know, seems a sweeping statement to make, and the one making it may be looked upon, especially by some of the older and more conservative men, as too much of an enthusiast. But my observations of this work, extending over a number of years, my contact with men of known ability and integrity, and my own experience during the past few years, convince me that such a statement will be fully verified as time goes on.

The use of porcelain was first resorted to from an esthetic point of view, and if it had no other recommendation, it would still hold a firm place with those whose desire it is to keep as close to nature in their work as possible, and to refrain from mak ng mouths unsightly, and in some cases repulsive, from the display of gold. But while this esthetic feature of porcelain is as important as ever, instead of being the first consideration in its use, it may, in the light of tooth-preserving qualities possessed by it, be almost considered last; for the preserving of the teeth should ever be our first aim. Porcelain will, I truly believe, save the teeth better than any other material we now have. I am not well versed in chemistry, and may not be able to satisfactorily explain why this is so. I can only say that probably this preserving quality comes from environment brought about by the improved conditions of the teeth filled with porcelain, the adjacent teeth, and surrounding tissues. A properly-glazed porcelain filling is easier to keep clean than the most highly-polished gold filling; it does not tarnish and gather food as gold will, and it is not in the least irritating to mucous membrane surfaces. These combined conditions tend to much greater cleanliness and consequent immunity from decay. Porcelain, by possessing such qualities, seems to invite the environment we look for in a clean, healthy mouth. Next to the preservative qualities of porcelain, I would place its compatibility to tooth structure. This quality cannot be too strongly commended, for it possesses it in a far greater degree than any other material. A tooth that is persistently uncomfortable under a metal filling, sensitive to thermal changes, and sometimes even to the apparently normal temperature of the food, will be perfectly comfortable with a porcelain filling. I think a reasonable inference to be drawn from this is, that with the use of porcelain many pulps may be kept alive that would have to be destroyed, or would die from irritation under metal fillings. The compatibility to tooth structure is probably the strongest feature in favor of porcelain.

A few words now of the esthetic feature, viz: Its harmony with the natural teeth in color and texture. Because I speak of this last it is not by any means that I consider it the least. This feature should of itself alone appeal to every one with the least artistic nature, and it is now I believe so well known by those even who are ignorant of its other qualities that it is hardly necessary for me to comment upon it. Even those who doubt the practicability of porcelain as a filling material cannot deny its esthetic value, its close approximation to the natural teeth.

The saving of fatigue to both patient and operator is a considerable factor in its favor, for though the time required to make a filling may take as long or longer than the insertion of a gold filling, the work is of a different kind, part of it being done out of the mouth. We all know the advantage this must be with nervous patients, to say nothing of the saving to the operator's back. I could take up the entire space of my paper detailing the many advantages possessed by porcelain as a filling material, and demonstrate why it is an ideal material to save teeth with, for it can be used in nearly all classes of cavities and under all conditions, and its harmony in results is most gratifying. I do not place it on a level with gold, but above it, as it will not only save all teeth that gold will save, but it will save teeth that gold in some cases cannot save. Facts in clinical experience have, I think, fully demonstrated this. Though I made the statement that I believed the use of porcelain was only limited by the operator's ability to successfully apply it, I do not by any means advise the beginner, or even the man of very limited experience, to at first attempt its use farther back than the second bicuspid, and it would be even better for a time to confine its use to the twelve anterior teeth. Its more extensive application will naturally come with experience. Before describing the method employed in making fillings, perhaps I should say a few words regarding the material I use, which is a high-fusing porcelain. I started to use it because of the peculiar qualities of porcelain itself, and the method of using it in the construction of fillings seemed to me to promise the best results. I do not say that results as good can not be obtained from a low-fusing porcelain, but I can say that though I have seen work done by experts in low-fusing and high-fusing porcelain, I have not yet seen any fillings made of low-fusing porcelain that can compare with the high-fusing for life-like color, perfection of contour or accuracy of adaptation.

The method of constructing a filling, which I shall give in detail, is not original with myself, but follows along the lines of some of those who have been most successful in the work. Some of the ideas are original with Dr. W. T. Reeves, of Chicago, to whose teaching I am greatly indebted for much of my knowledge in advanced methods in this work, and who, I believe, is justly rated one of the most expert porcelain workers in practice. The preparation of the cavity is the first step, and it is a comparatively simple operation. Of course, all decay must be removed, and cutting proceeded with until sound enamel supported by dentine is reached. When shaping of cavity is completed, there must be no undercuts of any kind, so, if in removing the decay, it is so shaped that there are undercuts they must be filled up with cement, and the cavity reshaped as though no such condition existed. There are no cavities in this work where self-locking forms or retaining walls are required. In fact, they are to be strictly avoided, or it will be impossible to remove the matrix without changing its form.

In approximal cavities in the anterior teeth, extending to the occlusal edge, the palatal wall is cut in excess of the labial, as the latter should aid in resisting the outward force exerted in mastication. In such a cavity, a broad base at the gingival aspect should be made, which is a seat for the filling. A form of seat must be made in all cavities, generally in the form of a triangle, which gives the filling a decided base to rest on, and also prevents it from slipping out of place when setting it. It is not retention, but simply a seat.

It is not necessary to cut cavities too deep, nor, on the contrary, must they be too shallow, or the cement will show through the filling. Never make a cavity perfectly round, and always avoid straight lines in cutting the margins. Slightly-curved lines show the joint much less, and are more pleasing to the eye. There must be no bevel to the margins; they must be well defined, perfectly even, smooth, and polished wherever possible. One of the main secrets of success in the work consists in having perfect margins. In shaping of cavities, I use round burs and small gem points, and for finishing the margins, sand-paper and fine cuttle-fish disks, Arkansas stone points, and sometimes dull plug finishing burs revolving very rapidly. If the rubber dam is used in preparing cavities, the color of the teeth should be got before applying it, as when it has been on the teeth a few minutes, they dry out and become lighter in color, and will mislead when you come to match the color for the filling. In approximal cavities, sufficient space must be obtained before starting. Sufficient space means a little more than would be required to fill the same cavity with gold. Nothing will handicap the work more than lack of space, and this must be kept well in mind. In labial and buccal cavities, wherever it can be done, the gum should be pushed out of the way with gutta-percha. Always get all the room possible in which to work. We will suppose we have a cavity in a central incisor, an approximal cavity, extending from well up toward the gum down to and including the incisal edge. It is already prepared, so we commence to make the matrix by taking a piece of platinum 1000 of an inch in thickness, a piece large enough to extend well beyond all the margins of the cavity, care being taken not to allow it to extend too far over the cervical margin. A piece of wet cotton is then used to depress the platinum into the cavity, which is done either with a round-nose burnisher, or a pair of round-nosed tweezers. As the platinum is carried into the cavity, care must be taken not to push the burnishing instrument through the cotton and platinum. At this stage of the work, do not attempt to turn the platinum over the margins, but pack with the cotton into cavity as thoroughly as possible. When it is removed, it will be found that the margins are quite well defined. Before returning it to the cavity, trim well away from the cervical margin, so that it will not press on the gum and prevent it from going up to place in subsequent burnishings. Return platinum to cavity for second step in the process, which consists of taking a piece of twilled tape, passing it up so as to engage all portions of the platinum, and drawing it in a direction that will cause it to lap all the margins at the same time; this distributes the folds equally around all the surfaces of the tooth, and prevents buckling badly in any one place. When the cavity is a very large one, the matrix may be packed with cotton, not allowing it to envelop the margins. When the platinum is removed from cavity, see if any more trimming of margins is necessary to allow free withdrawal after the final burnishings. Do not trim any more than is necessary, for the more platinum overlapping margins of the cavity, the easier it is to carry out the next stage of the work, which is done by holding the matrix in place with the fingers while all the wrinkles of the edges are burnished out, and it is thoroughly burnished home at every point, always burnishing from margin toward center. Leaving as much platinum overlapping the margin of cavity as possible facilitates building up and contouring the porcelain, as we then have the shape and size of the tooth reproduced in platinum, and it also stiffens the matrix. Before removing matrix from cavity this time burnish as smooth as possible. Of course, perforation of the matrix in burnishing is to be avoided as much as possible, but if we do find a small hole or tear there, it will cause no harm, for the porcelain body will br dge over if not used too wet. If there is any spring in the matrix after this burnishing, it will be overcome by the final step, which is done by taking a piece of heavy rubber dam, drawing tightly over all the margins of cavity with matrix in place, and burnishing over the rubber. With this method of holding, movement of matrix is impossible, as it is held in contact at every point.

During the fitting and burnishing of the platinum, it should be frequently annealed to keep it as soft as possible. To remove matrix from cavity after this final burnishing, gently tease it out with a fine exploring point, using great care so that its form may not be changed. If the cavity has been properly prepared, and there is no binding of tooth where platinum overlaps margins, the removal should be easily accomplished, though moisture with the perfect contact occasionally causes it to stick a little. Sometimes a current of air sent in at some accessible point will bring it out very nicely. After getting color of teeth and putting temporary stopping in cavity, the patient may now be dismissed, unless it is desired to finish at one sitting. It is in this matter of getting colors that we are called upon to exercise our artistic sense and nice discrimination in seeing shades and colors. I follow Dr. Reeves' system entirely in getting colors by building up the filling in layers, and though it seemed rather difficult at first, I would not under any circumstances go back to the system employed by the majority of porcelain workers of baking the filling in one solid color. It does not matter how near one comes in matching the tooth, the natural translucent effect so necessary can not be obtained when a solid color is used. Every tooth has more than one color or shade, which can best be described by calling them underlying colors. A tooth that at first sight appears to be just one color will, when closely examined, be seen to have two or three or more underlying colors, and to get the effect we are striving after, these colors must appear in the filling. This can only be done by baking the different colors in layers. It may seem almost impossible at first to see these different colors in the teeth, but you will be surprised when you have practiced looking for them, how in a short time you will begin to easily detect them. We will take a central incisor such as I am describing this filling for, for instance, and we will probably find that the neck of the tooth is yellow, about the middle third three is gray underlying the yellow, and the occlusal third or quarter finishes off in a gray, or blue over gray, and quite translucent. A filling of one solid color here would not blend at all, and it is just in this portion of the work where so many fail in bringing about the natural artistic effect that porcelain gives so well when proper methods are employed. The building up in layers helps to do away with the shadow problem; by breaking up the rays of light, it gives translucency, and prevents, in a large degree, reflection of the cement—three very important points. So we must look carefully for the underlying colors, noting their depth and intensity. The colors are in the dentine only, and reflect through the enamel, which is almost colorless and nearly the same in all teeth. It is not necessary at any time to mix two or more colors to get her to get a given shade. The different shades of any one color are produced by the varying depth of the layer used So, to produce your filling with the underlying colors, bake these colors in layers, baking each separately, and varying depth of layer according to depth of shade. When you have built up to nearly full contour, lay on a neutral color or enamel, and the finished filling will show the underlying colors reflecting through the enamel. I know of no other way in which the translucent effect can be obtained. I will briefly describe the building up and baking of filling for this cause. The first baking, which generally forms about one-third of filling, would be of a yellow foundation body, which fuses at a higher degree of heat than the material of which the rest of the filling is made. After this baking, I put on the yellow, gray and blue, in their respective places, allowing them just to blend together by a gentle agitation. When this is baked, the shrinkage will probably require more of these colors, but as the gray at the end of the tooth is over the blue, I would only use yellow and gray for this layer. After this baking, a thin layer of yellow over all may be used, followed by a neutral color, or it may only require the neutral color, according to the intensity of yellow. The color effect of a filling made in that way harmonizes almost perfectly with the tooth and its surroundings, and is hard to distinguish from nature's work itself. Where deeper colors or effects are required, or a stain in the tooth must be carried out in the filling, it can be produced by what are known as primary colors, which, though they are mixed with oil, and laid on like paints, are really high-fusing bodies. A set of these colors is invaluable in a porcelain outfit, and nature can be imitated so closely that it is almost impossible to detect their use.

The labial portion of the tooth is nearly always yellow, and a porcelain filling there would only show yellow on the surface, though it would be built up in layers the same as the other. Should the cavity extend well down the tooth, it would, of course, be necessary to blend in the filling whatever variation in color the tooth showed. One of the main points to be kept in mind in building up in layers, is, that the depth or intensity of any given color is governed entirely by the thickness of the layer of that particular color.

The length of my paper will hardly permit me to go into details of fusing. An electric furnace is, of course, best where the electric current is available, but I believe there are gasoline furnaces in the market now that are giving very satisfactory results. One must become familiar with the use of any furnace, and understand the characteristics and fusing points of the porcelain bodies he is using before attempting practical work.

In contour work, before stripping the platinum from the filling, it should be tried in the mouth, when if it is too full it may be ground off and put in the furnace and reglazed. It is a good plan to try in the cavity before putting on the final layer, to see if it is too full or not full enough. There is one thing I must warn you against when trying in for this purpose, don't try and match your color at the same time, for the platinum overlapping the tooth gives it a bluish cast, and makes it appear much darker than it really is.

After the final baking, the platinum is removed, and the cavity side of the filling etched with hydrofluoric acid to hold the cement. It is now ready for setting, and here is the part of the work where so many fail. The usefulness of many a perfect-fitting filling is destroyed from faulty manipulation in its setting. First, it must be set under pressure, for it is the perfect adaptation of the filling, plus being set under pressure, that retains it in place. Undercuts, any device of cavity formation termed interlocking against lateral strain, or pins baked in the filling, are wholly unnecessary, for clinical experience has proven that close adaptation in all parts of the cavity with the cement setting under pressure, will retain fillings in any position they may be placed: Here, then, is where strict rules must be observed or failure will surely follow. Whenever possible, the rubber dam should be applied, and in setting fillings in approximal spaces, wedges are used to retain them in place until the cement is hardened. I use a wedge of the right thickness made from a stick of orange wood.

The Harvard cement is, I believe, the best adapted for this work; it does not set too quickly, is very sticky, and comes in a large variety of colors. The color of the cement should match the tooth as closely as possible. Having the rubber dam on, and the filling where it can be readily picked up, mix the cement to a thick creamy consistency, cover the entire surface of the cavity, gently press filling into place, being sure that it is in place, and at once insert the wedge, so that it will hold it with a firm, steady pressure. It should so remain from twenty to thirty minutes. The surplus of cement may then be trimmed off, and if the work has been properly done, it is finished. I have noticed that nearly all porcelain-filling workers, when writing or speaking on the subject, tell us that the margins are to be finished at a subsequent sitting, with sand-paper, cuttle-fish disks, etc. If the margins of the cavity are properly prepared, and care is used to build the porcelain perfectly to the margins on the matrix, you will have a filling that fits flush, and does not require any finishing. Porcelain workers and the manufacturers of the enamel bodies speak of being able to grind the porcelain and then polish it, and so you can, but when it is done it is doubtful if it is equal to the glazed surface as it comes out of the furnace. The occlusal surface of a filling may be ground if necessary, so that it will be kept clean by friction, but the grinding of all other surfaces should be avoided as much as possible. When it is impossible to apply the rubber dam, and moisture by other means can not be kept away as long as desired, I flow hot paraffine over the surface to protect it as long as possible.

Although making porcelain fillings for labial cavities might appear to be more simple than those for any other position, such is not the case. The building up of the porcelain in the matrix is easier and takes less time than this part of the work for cavities in other positions, but the forming of the matrix for labial cavities, especially where they extend beneath the gingival margin, is more difficult than it is in cavities on the surface of the teeth where the tape and rubber dam can be used as described. Cavities extending from the labial to the approximal surface are perhaps some of the most difficult we have to contend with. In forming the matrix for labial cavities, a piece of platinum as large as consistent should be used, to facilitate handling and to give it more stability, and so prevent springing. In such cases, I tie the matrix in place with floss silk, forming a loop, bringing the ends to the front and securing with a half surgeon's knot. To remove spring, a compress of wet cotton may be used to press down all portions of matrix into place, or a piece of soft rubber pressed home by the concaved end of a piece of wood is quite effective. The tape and rubber dam should always be used when possible, as it is a wonderful aid in the work. Patience and persistence will overcome in these cases, as in all others. With small fillings for labial cavities, it is not easy to tell at a glance the position in which they should be placed, so it is a good plan to make a small mark on the filling, indicating the correct position.

When the cement is mixed and placed in cavity, you want to be able to pick up the filling and place it at once. There is no time to study which end should be nearest you, or *vice versa.*

Remember, the cement will darken your filling a trifle no matter how near it may match color of tooth, so the filling should be just a shade lighter before it is set. When the filling is finished, before setting it, it is a good idea to put it in cavity, and let your patient look at it, because when it is in the tooth that way without cement, and the saliva softening the effect, it looks so natural that frequently it can not be detected, and the patient is delighted. First impressions, you know, are lasting, and if the patient's first sight of the filling is after it is cemented in, with the teeth bleached two or three shades lighter than their natural color, and the line of cement showing around the margins, the first impression might not be so pleasing, though in a few days with the teeth the natural color and the cement gone, the filling blends with its surroundings, and looks very natural and life-like.

Though I have endeavored to set this subject before you in simple language, and in as plain a manner as possible, I am aware that the process described may seem complicated and I almost regret that I could not have given a practical demonstration of the work, for that which we see always seems more simple, and leaves its impress on us more clearly than that which we hear.

There is much more that I could say, but time will not permit and this field is so wide a one that one paper could not possibly cover it in a satisfactory manner.

